# The Influence of Early Gymnastic Exposure on the Triangular Fibrocartilage Complex in the Adolescent Wrist

**DOI:** 10.1016/j.jhsg.2026.100969

**Published:** 2026-02-24

**Authors:** Anne-Sophie van der Post, Sjoerd Jens, Laura S. Kox, Miryam C. Obdeijn, Roelof-Jan Oostra, Mario Maas

**Affiliations:** ∗ Amsterdam UMC location University of Amsterdam, Radiology and Nuclear Medicine, Meibergdreef 9, Amsterdam, the Netherlands; † Amsterdam Movement Sciences (AMS), Sports and Musculoskeletal Health, Amsterdam, the Netherlands; ‡ Rijnstate Hospital, Radiology and Nuclear Medicine, Arnhem, the Netherlands; § Amsterdam UMC location University of Amsterdam, Plastic, Reconstructive and Hand Surgery, Meibergdreef 9, Amsterdam, the Netherlands; ‖ Amsterdam UMC location University of Amsterdam, Medical Biology, Clinical Anatomy and Embryology, Meibergdreef 9, Amsterdam, the Netherlands

**Keywords:** Adolescent, Gymnastics, Magnetic resonance imaging, Triangular fibrocartilage, Wrist injuries

## Abstract

**Purpose:**

This study aimed to evaluate triangular fibrocartilage complex (TFCC) appearance on magnetic resonance imaging (MRI) and the correlation between central triangular fibrocartilage (TFC) thickness and ulnar variance (UV) in adolescent asymptomatic gymnasts.

**Methods:**

This retrospective analysis of a prospective cohort selected 12–18 years old asymptomatic gymnasts and healthy controls from the Physeal MRI study, which is a single-center study on physeal injury that included gymnasts with wrist pain, asymptomatic gymnasts and healthy nongymnasts from June 2015 until November 2017. A standardized scoring form was used for assessment of TFCC morphology on MRI. Bone age and UV were determined on radiographs. TFCC morphology was assessed on 3T MRI and categorized. Statistical differences between groups were calculated using a chi-square test or Fisher exact test. Spearman’s correlation coefficient was calculated between central TFC thickness and calendar/bone age, UV, height, and weight. Correlations were interpreted as poor (≤0.20), fair (0.21–0.40), moderate (0.41–0.60), substantial (0.61–0.80), and excellent (0.81–1.00). Multiple linear regression was performed to determine predictors of central TFC thickness.

**Results:**

Forty-one adolescents (23 nongymnasts, 18 gymnasts, median age 14 years, 21 female) were included. No differences were found in TFCC appearance. Correlations between central TFC thickness and UV were substantial in both groups. Multiple linear regression analysis showed that UV and bone age together were significant predictors for central TFC thickness in nongymnasts (*P* < .001). However central TFC thickness could not be predicted by UV and bone age in gymnasts.

**Conclusions:**

Early gymnastic exposure does not influence TFCC appearance on MRI. However, a misbalance between central TFC thickness and UV appears to exist for adolescent gymnasts and this should be taken into consideration when assessing gymnastic wrist injury.

**Type of study/level of evidence:**

Diagnostic III

The triangular fibrocartilage complex (TFCC) is interposed between the distal ulna and ulnar carpus and acts as a stabilizer of the distal radioulnar wrist joint and axial load distributor.^1^ Although TFCC lesions were previously considered to occur exclusively at a skeletally matured age, an increasing number of cases are reported in skeletally immature gymnasts.^2^, ^3^, ^4^ Radial, central, ulnar, and volar TFCC tears as well as thinning of the central triangular fibrocartilage (TFC, also known as articular disc) are reported.^2^, ^3^, ^4^ Commonly, this injury is associated with repetitive wrist movements and ulnar abutment in a wrist with a positive ulnar variance (UV) (ie, a relatively longer ulna than radius).^5^^,^^6^ Premature growth arrest of the distal radius, known as “gymnasts wrist”, may cause positive UV because of premature arrest of the growth plate of the radius in skeletally immature gymnasts compared to nongymnasts.^4^^,^^7^, ^8^, ^9^, ^10^, ^11^

In healthy adults, the ulna bears around 20% of the total axial load in which the articular disc distributes around 12% of the axial load from the radius to the ulna.^12^ Similar distributions account for healthy adults with positive and negative UV.^13^ This is explained by the negative correlation between UV and central TFC thickness, a more positive UV correlates with a thinner central TFC and vice versa.^12^^,^^14^, ^15^, ^16^, ^17^ Skeletally immature gymnasts are believed to have higher axial loads on the radius because of their physiologically negative UV making their radial physis more prone to injury.^7^^,^^11^^,^^18^, ^19^, ^20^ Yet, in healthy nongymnast adolescents a similar negative correlation has recently been demonstrated.^21^ If, however, the change in TFC thickness during maturation does not occur exactly synchronized with the change in UV in gymnasts, ie, a misbalance between central TFC thickness and UV, changes in axial load distribution may occur. It could be hypothesized that the additional load on the TFCC may cause TFCC injury.

The effects of gymnastic exposure on the TFCC in skeletally immature athletes remain unknown. The aim of this study therefore is to detect potential early differences in TFCC appearance on magnetic resonance imaging (MRI) between skeletally immature asymptomatic gymnasts and healthy nongymnasts. Additionally, central TFC thickness and the correlation between central TFC thickness and UV will be compared in order to detect a potential misbalance as a possible explanation for gymnasts to develop TFCC injury.

## Materials and Methods

### Study design

This observational cross-sectional study was based on the Strengthening the Reporting of Observational Studies in Epidemiology guidelines for reporting observational studies.^22^ It retrospectively analyzed prospectively collected study data from the Physeal MRI study from June 2015 until November 2017 at the Amsterdam University Medical Center.^23^ The Physeal MRI study was a prospective cohort study on physeal injury that included gymnasts with radial sided wrist pain, asymptomatic gymnasts and healthy nongymnasts between 12 and 18 years old. The present study included only the asymptomatic gymnasts and the healthy nongymnasts. The Physeal MRI study was approved by the institution’s Medical Review Ethics Committee (reference no. 2014_382) and was performed according to the Declaration of Helsinki.

### Participants

Inclusion criteria for the asymptomatic gymnasts were no history of wrist pain in the past 6 months and participation in gymnastics for at least 1 year, with discontinuation of gymnastics no more than 6 months prior to inclusion. Inclusion criteria for the healthy controls were nongymnasts who did not have wrist pain over the last 6 months and did not participated in gymnastics or performed wrist loading sports over 2 times a week. Both groups were recruited at gymnastic clubs through the bring a friend strategy and notifications at our institution. Exclusion criteria for both groups were insufficient MRI quality, fused distal radial physes, diagnosed growth disturbance, musculoskeletal diseases, a history of wrist fracture, surgery or infection. Written informed consent was given by each participant as well as the parent or legal guardian. Demographic information of all participants was extracted from questionnaires filled out by the participants. Extracted information included calendar age, sex (boy or girl), side of included wrist, gymnastic participation, starting age with gymnastics, other sport participation and sport intensity.

### Imaging

First, participants underwent a conventional posteroanterior radiograph following a standardized protocol with 90° shoulder abduction, 90° elbow flexion and neutral forearm position (focus detector distance 1.30 m) of one randomly assigned wrist. Then, participants underwent an MRI of that same wrist, using a 3T MRI scanner (Ingenia, Philips Healthcare, Best, The Netherlands) in a feet first, supine position with the wrist placed neutral, alongside the body, using a dedicated wrist coil (eight channel, receive only).

The included MRI sequences for TFCC assessment were a turbo spin-echo (TSE) proton-density (PD) weighted sequences in three planes without fat saturation (1,500–2,000 ms repetition time (TR), 20 ms echo time (TE), 90° flip angle, 1.5–2.5 mm slice thickness, 0.30 × 0.30 mm spatial resolution, 4:06–4:39 minutes scan time) a TSE PD weighted coronal sequence with fat saturation by spectral attenuated inversion recovery (SPAIR) (2000 ms TR, 30 ms TE, 90° flip angle, 2.5 mm slice thickness, 0.30 × 0.32 mm spatial resolution, 4:12 minutes scan time) and a TSE T2 weighted axial sequence with fat saturation by SPAIR (3001 ms TR, 60 ms TE, 90° flip angle, 2.5 mm slice thickness, 0.30 × 0.36 mm spatial resolution, 4:58 minutes scan time).

### Image assessment

Radiographs as well as MRIs were anonymized and the observers were blinded for participant groups (ie, gymnasts or nongymnasts). The order of image assessment was randomized and UV was determined in a separate session than TFCC assessment. Because discrepancies between skeletal age and chronological age among youth athletes emphasize the importance of considering skeletal maturity when evaluating young athletes, bone age was determined using the validated BoneXpert software (v2.0.1.3; Visiana, Holte, Denmark).^24^ Ulnar variance was assessed by one dedicated musculoskeletal radiologist (S.J.) using the recommended adapted perpendicular method.^25^

The TFCC morphology, including assessment of wrist position on axial images, was assessed on MRI in consensus by two dedicated musculoskeletal radiologists (M.M. and S.J.) with 26 and 1 years of experience, respectively, on a diagnostic PC workstation with high resolution monitor using IMPAX software version 6.6.1.4024 (AGFA HealthCare N.V., Mortsel, Belgium). Systematic assessment was performed according a scoring form that was developed in a prior study by van der Post et al^26^ (2021) (Fig. S1 available online on the Journal’s website at https://www.jhsgo.org). A 15-minute break was held after assessment of every 10 participants. The observers were allowed to use the window and zoom functions. Central TFC thickness was measured in consensus by the same two observers, using the midcoronal slice where the central TFC appeared the thinnest and measuring the shortest distance between the two articular surfaces (Fig. S1). Any unexpected abnormalities not related to the study objectives were classified as incidental findings. Incidental findings considered potentially clinically significant were documented.

### Statistical analysis

Descriptive statistics and central TFC morphology categories were illustrated as numbers or medians (interquartile ranges [IQRs]). Statistical differences between groups were calculated using a chi-square test for ordinal data, a Wilcoxon rank sum test for skewed continuous data, and a Fisher exact test for categorical data with small cell count (TFC morphology). The analysis of covariance was used to control for potential confounding variables.

Spearman’s correlation coefficient (ρ) with 95% confidence intervals (CIs) between central TFC thickness and continuous variables were calculated. TFC thickness measurements that were not possible because of a hyperintense signal intensity disrupting the disc (four nongymnasts and two gymnasts) were excluded from all analyses. All correlation coefficients were interpreted based on the interpretations by Landis and Koch as poor (≤ 0.20), fair (0.21–0.40), moderate (0.41–0.60), substantial (0.61–0.80), and excellent (0.81–1.00).^27^ Statistical differences in correlation coefficients between groups were calculated using the Fisher r-to-Z transformation. Potentially associated variables with the central TFC thickness (correlation coefficients with a *P* value < 0.20) were included in multiple linear regression analysis with backward stepwise elimination. *P* values < 0.05 were considered significant.

## Results

### Patient demographics

A total of 41 adolescents (23 nongymnasts and 18 gymnasts) were included in the present study with a median calendar age of 14 years (Table 1). One nongymnast was excluded based on insufficient magnetic resonance image quality because of major motion artifacts. Between nongymnasts and gymnasts, no statistical difference existed for calendar age, weight, sex, and side of the assessed wrist.

**Table 1:** Patient Characteristics

Variables	Nongymnasts (n = 23)	Gymnasts (n=18)	Difference *P* value
Calendar age (y)			
Median (IQR)	13.5 (12.6–14.5)	14.0 (13.6–15.2)	.15
Bone age (y)			
Median (IQR)	13.4 (12.5–14.2)∗	12.0 (11.5–13.6)∗	.04
Sex, (no.)			
Girls/boys	12/11	9/9	1.00
Side (no.)			
Right/left	13/10	12/6	.74
Height (cm)			
Median (IQR)	165.5 (160.2–171.8)∗	158.6 (154.2–164.8)∗	.02
Weight (kg)			
Median (IQR)	53.0 (43.0–61.5)	47.0 (42.3–50.0)	.21
Sport type			
Sport (no.)	Field hockey (7)	Gymnastics (18)	NA
	Netball (5)		
	Soccer (2)		
	Multiple (5)		
	Other (4)		
Sport intensity (h/week)			
Median (IQR)	3.0 (2.6–5.0)∗	15.8 (12.1–33.3)∗	<.001
Start age gymnastics (y)			
Median (IQR)	NA	6.0 (5.3–6.8)	NA
Gymnastic experience (y)			
Median (IQR)	NA	8.5 (6.7–10.3)	NA
Gymnastic training level (no.)	NA	Elite (n = 16) Nonelite (n = 2)	NA

NA, not applicable.

∗ Indicates a statistically significant difference.

### MRI appearance TFCC

**Figure 1:**
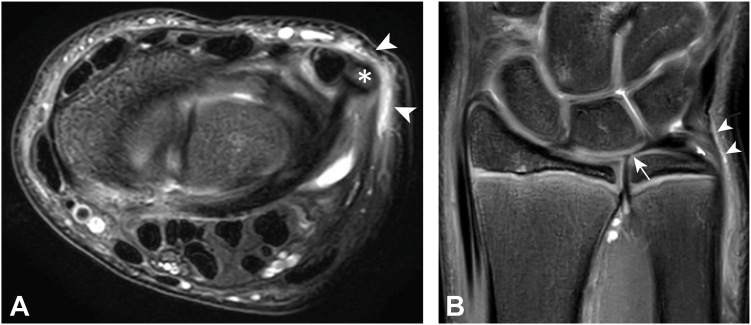
T2-weighted fat saturated MRI of the same asymptomatic gymnast showing subcutaneous fat infiltration (arrow heads) surrounding the ulnar styloid process (asterisks) suggesting a sort of ulnar wrist impingement. **A** Axial T2-weighted fat saturated MRI. **B** Coronal T2-weighted fat saturated MRI. Note the vertical linear increased signal intensity within the articular disc (arrow), which was observed in nongymnasts as well as asymptomatic gymnasts.

**Figure 2:**
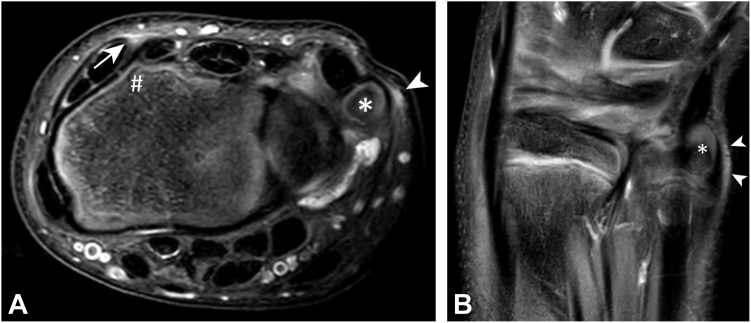
T2-weighted fat saturated MRI of the same asymptomatic gymnast with high signal intensity within subcutaneous fat on the dorsal wrist (arrow) surrounding lister’s tubercle (hashtag) of the distal radius and on the ulnar side (arrow head) of the styloid process (asterisks) suggesting a sort of dorsal as well as ulnar wrist impingement. **A** Axial T2-weighted fat saturated MRI. **B** Coronal T2-weighted fat saturated MRI.

No statistical differences were found in MRI appearance of the individual TFCC components (Table 2). As an incidental finding, traumatic high signal intensity subcutaneous fat infiltration was found more frequently in gymnasts than in nongymnasts on the ulnar side of the wrist (13 vs 4 respectively, *P* = .001) and/or on the dorsal side of the wrist (12 vs 2, respectively, *P* < .001; Figs.1 and 2).

**Table 2:** MRI Characteristics Scored According to the Score Form (S1). Adapted From van der Post et al^26^ (2021) in Frequencies of Observations for all Participants for Nongymnasts and Gymnasts

Scoring Item	MRI Characteristics	Nongymnasts (n = 23) No.	Gymnasts (n = 18) No.	Difference *P* value
Triangular fibrocartilage				
Coronal morphology	Slightly radially tilted bowtie	11	10	.30
	Shorter, thicker and more horizontal	11	5	
	Thinner and more stretched	1	3	
Coronal homogeneity	Homogeneous hypointense	10	5	.48
	Diffuse increased SI	9	11	
	Vertical linear increased SI	4	2	
	Other	0	0	
Sagittal morphology	Symmetrical biconcave	13	7	.10
	Dorsal thicker than volar	9	6	
	Volar thicker than dorsal	1	5	
Dorsal radioulnar ligament				
Sagittal morphology	Continuous with TFC SI	20	18	.50
	Discontinuous with TFC SI	1	0	
	Not able to assess	2	0	
Axial fiber continuity	Continuous fibers	23	15	.08
	Fiber discontinuation	0	2	
	Not able to assess	0	1	
Volar radioulnar ligament				
Sagittal morphology	Continuous with TFC SI	16	13	.64
	Discontinuous with TFC SI	5	5	
	Not able to assess	2	0	
Axial fiber continuity	Continuous fibers	23	17	.44
	Fiber discontinuation	0	1	
	Not able to assess	0	0	
Proximal lamina				
Coronal homogeneity	Homogeneous hypointense	7	3	.62
	Diffuse lamination	14	13	
	Not able to assess	2	2	
Distal lamina				
Coronal homogeneity	Homogeneous hypointense	1	1	1.00
	Diffuse lamination	21	17	
	Not able to assess	1	0	
Ligamentum subcruentum				
Coronal morphology	Hyperintense signal between lamina	17	14	.81
	Not visible	6	4	
Meniscus homologue				
Coronal morphology	Diffuse hypointensity	18	15	.68
	Clearly delineated hypointensity	3	3	
	Not visible	2	0	
Wrist position				
Axial position	Supination	14	10	.86
	Neutral	8	8	
	Pronation	1	0	
Extensor carpi ulnaris tendon				
Axial position	Completely within groove	13	13	.56
	Partially within groove	7	4	
	Completely outside groove	3	1	
Axial peri-tendinous SI	Hypointense/intermediate	4	1	.29
	Focal increased	19	17	
Location	Proximal from styloid process	15	16	.61
	Distal from styloid process	3	1	
	At styloid process	1	0	
Axial intratendinous SI	Homogeneous hypointense	12	5	.20
	Focal or linear increased	11	13	
Location	Proximal from styloid process	4	5	1.00
	Distal from styloid process	5	6	
	At styloid process	2	2	
Joint effusion				
Prestyloid recess	Conical shaped	8	4	.13
	Tubular shaped	1	6	
	Saccular shaped	4	3	
	Not visible	15	5	
DRU joint effusion	Absent	4	2	.84
	Small amount	18	15	
	Substantial amount	1	1	
PT joint effusion	Absent	5	3	.19
	Small amount	10	13	
	Substantial amount	8	2	
Ulnar sided cysts	Absent	22	13	.07
	Present volar	8	5	
	Present dorsal	0	0	
Other findings				
Dorsal impingement	Absent	21	6	<.001∗
	Present	2	12	
Ulnar impingement	Absent	19	5	.001∗
	Present	4	13	
Other	Absent	15	7	.12
	Present	8	11	

DRU, distal radioulnar; PT, pisotriquetral; SI, signal intensity.

∗ Indicates a statistically significant difference.

### UV and central TFC thickness

**Figure 3:**
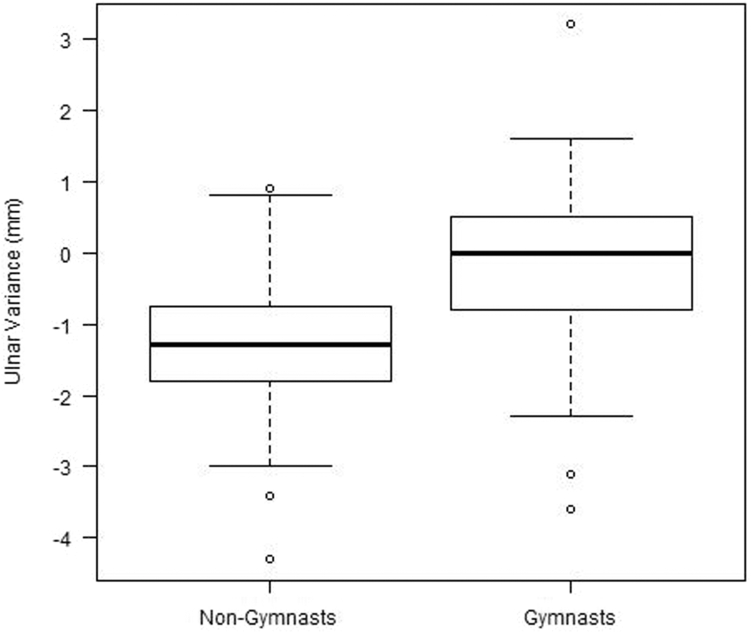
Box plot shows a statistically more negative ulnar variance in nongymnasts than in gymnasts.

**Figure 4:**
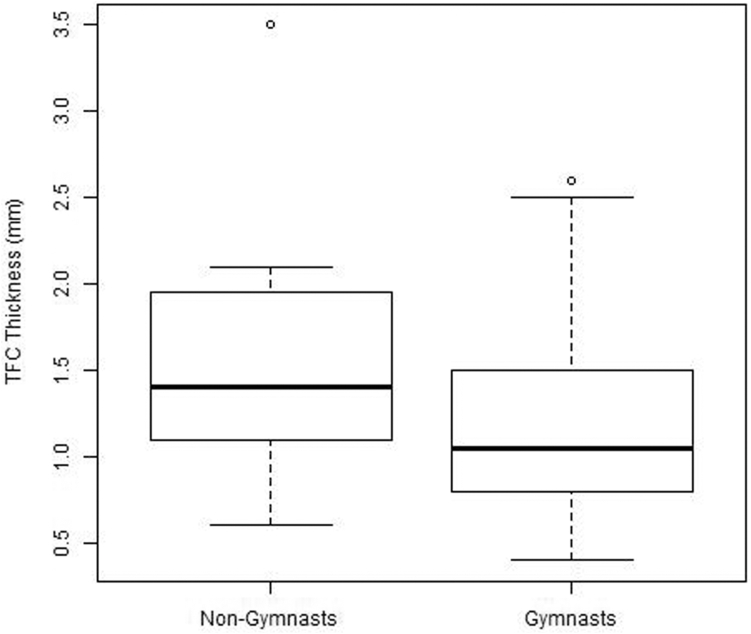
Box plot shows no statistical difference in central TFC thickness between nongymnasts and gymnasts.

Measurement outcomes of central TFC thickness and UV are depicted in Figures 3 and 4. Median UV was –1.3 (IQR = –1.8 to –0.8) mm for nongymnasts and 0.0 (IQR = –0.8 to 0.5) mm for gymnasts, which differed statistically (*P* = .01). A median central TFC thickness of 1.4 mm (IQR = 1.1–2.0) was found for nongymnasts and 1.1 mm (IQR = 0.9–1.5) for gymnasts, which did not differ between groups (*P* = .11). Even when corrected for bone age and/or height, central TFC thickness did not differ between both groups.

### Correlations

**Figure 5:**
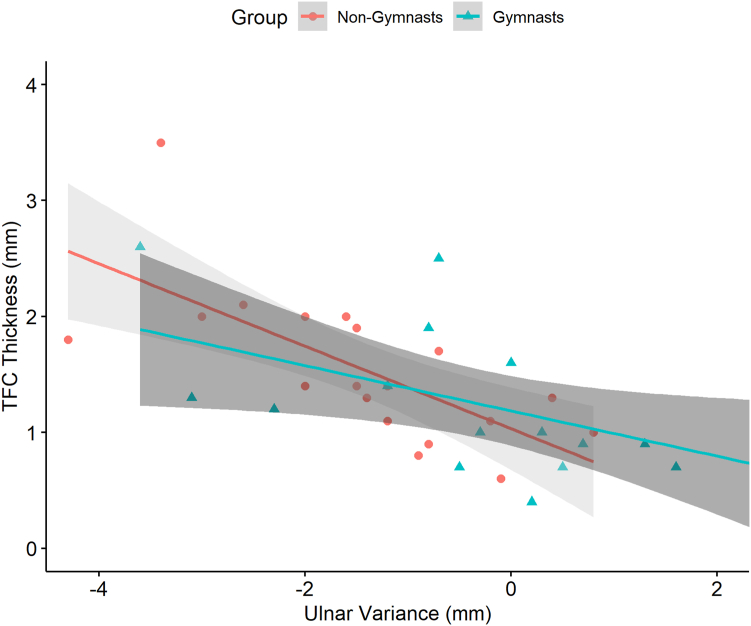
Scatterplot shows the substantial negative correlation between ulnar variance and central TFC thickness for both groups.

A substantial, negative correlation between central TFC thickness and UV was found in both groups, indicating that a decrease in central TFC thickness relates to an increase in (ie, more positive) UV and vice versa (Fig. 5). The correlation coefficient for nongymnasts was –0.80 (95% CI = –0.92 to –0.54, *P* < .001) and was –0.64 (95% CI = –0.86 to –0.21, *P* = .008) for gymnasts, without significant difference between both groups.

**Figure 6:**
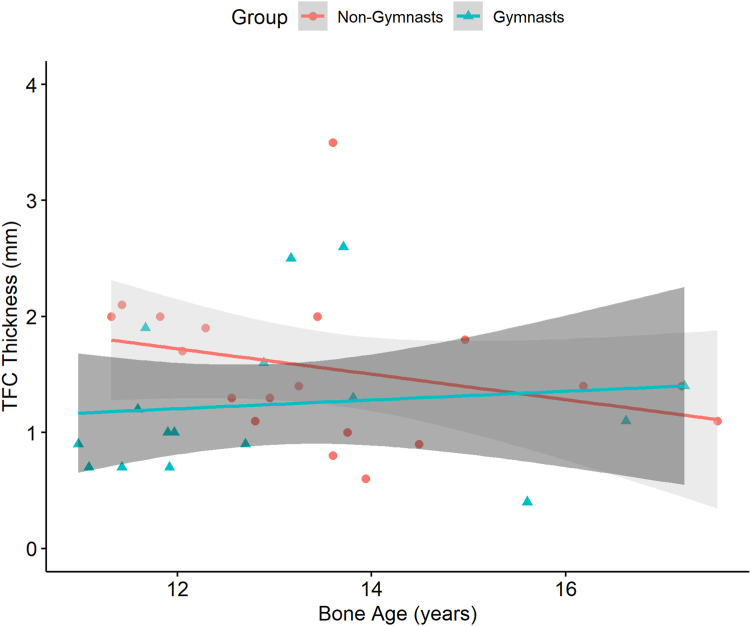
Scatterplot shows the moderate negative correlation between bone age and central TFC thickness for nongymnasts and the absence of a correlation for gymnasts.

A moderate, negative correlation was found between central TFC thickness and bone age for nongymnasts (ρ = –0.48, 95% CI = –0.77 to −0.04, *P* = .04), indicating that a decrease in central TFC thickness is related to an increase in bone age and vice versa (Fig. 6). No correlation between central TFC thickness and bone age was found in gymnasts (ρ = –0.36, 95% CI = –0.17 to 0.72, *P* = .17), which significantly differed from nongymnasts (*P* = .02).

No correlations were found between central TFC thickness and calendar age (ρ = –0.07, 95% CI = –0.51 to 0.39, *P* = .77), height (ρ = –0.22, 95% CI = –0.25 to 0.64, *P* = .38) or weight (ρ = 0.01, 95% CI = –0.44 to 0.46, *P* = .95) for nongymnasts nor between central TFC thickness and calendar age (ρ = –0.01, 95% CI = –0.50 to 0.49, *P* = .97), height (ρ = –0.14, 95% CI = –0.38 to 0.60, *P* = .60) or weight (ρ = –0.01, 95% CI = –0.44 to 0.46, *P* = 0.95) for gymnasts.

### Regression analysis

Ulnar variance and bone age were included in multiple linear regression analysis (Table 3). For nongymnasts, UV and bone age together predicted 51% (R^2^ = 0.51) of the variation in central TFC thickness (*P* = .001). For every millimeter increase in UV, corrected for bone age, a decrease in central TFC thickness of –0.35 mm can be expected (*P* < .001). For gymnasts, no overall significance was found for the regression model (*P* = .097). No correlation and therefore no multicollinearity existed between UV and bone age in both groups.

**Table 3:** Multiple Linear Regression Analysis With TFC Thickness as a Dependent Variable for Nongymnasts and Gymnasts

	Nongymnasts Adjusted R^2^ = 0.51∗		Gymnasts Adjusted R^2^ = 0.19	
Variable	β-coefficient (95% CI)	*P* value	β-coefficient (95% CI)	*P* value
Ulnar variance	–0.35 (–0.53 to –0.17)	<.001∗	–0.20 (–0.38 to –0.013)	.038∗
Bone age	–0.10 (–0.23 to 0.026)	.11	0.041 (–0.12 to 0.20)	.59

∗ Indicates a statistically significant difference.

## Discussion

The present study showed no difference in TFCC appearance on MRI between asymptomatic adolescent gymnasts and healthy adolescents, indicating no early detectable effects of gymnastic exposure. Even though UV was less negative in gymnasts than in nongymnasts and no difference was found in central TFC thickness, as shown in the boxplots in Figures 3 and 4, the negative correlation between UV and central TFC thickness was substantial in both groups. Bone age, however, was correlated with central TFC thickness only in nongymnasts. When UV and bone age were included in multiple regression analysis, central TFC thickness was only significantly predicted in nongymnasts and not in gymnasts. Compared to nongymnasts, central TFC thickness in gymnasts can no longer be predicted by UV when corrected for bone age, ie, a misbalance appears to exist for skeletally immature gymnasts.

Degenerative central communicating central TFC perforations have been reported as most frequent TFCC injury in young gymnasts.^20^^,^^28^ Yet, we found a vertically oriented linear increased signal intensity in the center of the central TFC in asymptomatic gymnasts as well as healthy adolescents. Additionally, we did not find a difference in early signs of central TFC degeneration such as a diffuse increased central TFC signal intensity.

The present study did find a 1.4 years lower bone age in asymptomatic gymnasts compared to healthy adolescents. A similar delay in bone age, ranging from 1.3 to 1.8 years, has been previously shown in elite female skeletally immature gymnasts.^29^, ^30^, ^31^ We hypothesize that this delay in bone age is responsible for the absence of a correlation between TFC thickness and bone age in asymptomatic gymnasts. This insinuates that central TFC thickness does not change on the same pace as bone age in gymnasts, probably because of repetitive axial loading from a young age, potentially putting them at risk for injury by altering axial load distribution over the radius and ulna.

A remarkable incidental finding was the high amount of gymnasts that showed subcutaneous fat infiltration on the dorsal and/or ulnar side of the wrist. This ulnar fat infiltration did not cause wrist pain and should therefore not be automatically interpreted as dorsal wrist (capsular) impingement. This phenomenon has not been previously reported and we hypothesize that the locations of fat infiltration might be explained by the use of hand grips, applying ulnar and/or dorsal pressure on the even and uneven parallel bars, or by contusional changes sustained during exercises.

### Clinical and research implications

Similar to bone age, UV is considered a dynamic phenomenon of maturation since it is usually negative in the skeletally immature and becomes more positive until physeal closure.^18^ Higher axial loads on ulnar negative wrists are believed to induce distal radial physeal injury, which may lead to a more positive UV and eventually impingement on the ulnar side of the wrist.^18^ Nevertheless, a statistical relationship between positive UV and distal radial physeal injury or wrist pain has not yet been demonstrated. Central TFC thickness also appears to be a dynamic feature during skeletal maturation as it becomes thinner with bone age and with a relatively longer ulna.^21^ Central TFC thickness, however, has systematically been ignored in the multivariate approach in search of the statistical relationship between positive UV and distal radial physeal injury or wrist pain.^7^^,^^8^^,^^18^

Distal radial physeal injury is considered to be the result of indirect injury of the blood supply.^32^ When the wrist is positioned in pronation, the ulna becomes relatively longer and axial forces on the ulna increase significantly.^13^ Repetitive axial loading, especially in pronation and ulnar deviation of the wrist such as in a cartwheel, might therefore cause similar indirect injury to the central TFC vascularity, accelerate central TFC thinning and cause a misbalance. It is known that newborns have higher percentages of peripheral vascularization of the central TFC disc.^33^

If the decrease in central TFC thickness is more rapid in gymnasts than the increase in relative ulnar length and therefore misbalanced, the combination of the variables UV and central TFC thickness together might be able to finally demonstrate a relationship with distal radial physeal injury or wrist pain. Consequently, symptomatic gymnasts might in fact be more vulnerable for developing distal radial physeal injury if original central TFC thickness was already thinner and/or decreases more rapidly in thickness, inducing higher axial pressures on the radial sides of the wrist. Therefore, it would be interesting to assess relations between UV, central TFC thickness and wrist pain in future studies on symptomatic gymnasts.

### Limitations

The major limitation of the present study was that a relatively small number of wrists were included. Statistical analysis may therefore be limited by low power and results should be interpreted as exploratory. The results highlight potentially meaningful effects worth investigating and future studies will be able to confirm or refute our conclusions. In addition, this prohibited further subanalyses on boys and girls or inclusion of additional variables in regression analysis, which could have provided valuable additional insights.

Another major limitation is the retrospective study design, analyzing data that was prospectively collected data for a slightly different study aim. Even though it was a deliberate decision not to include the symptomatic gymnasts from the MRI Physeal study because of the clinical suspicion of gymnast wrist characterized by radial sided wrist pain, this might have led to an inclusion bias. Yet, we believe that analyzing valuable existing data on a young asymptomatic population provides essential insights before analyzing symptomatic gymnast wrists.

Additionally, even though wrists were positioned in a neutral position in the wrist coil, the majority of both groups wrists were actually scanned in supination (Table 2). It is known that UV measurements change with pro- and supination.^34^ It is however unknown whether central TFC thickness measurement is influenced by wrist position and therefore potentially influences true correlations. The only prior study that analyzed the articular disc on MRI in different wrist positions did not find any measurable changes in coronal nor sagittal size.^35^ However, no central TFC thickness measurement was performed and the influence of wrist position remains unclear. The influence of this supinated position on actual central TFC thickness therefore remains unclear. It has been shown that the ulnar attachment appearance is influenced in pro- and especially supination.^35^ Hence, potential ulnar TFCC pathology could have been missed in our assessment.

Finally, measurement of true UV on radiographs is complicated by the possible variation in residual cartilage cap of the distal ulna. In the present study, we assume that this cartilage cap is comparable between both groups; however, it could be possible that variations between groups influence the correlations between UV and central TFC thickness.

### Conclusions

This extensive study on asymptomatic adolescent gymnasts showed no effect from early gymnastic exposure on TFCC appearance on MRI compared to healthy nongymnasts. The study did show that a substantial correlation exists between TFC thickness and UV in both groups. However, when corrected for bone age, TFC thickness can no longer be predicted by UV in gymnasts. Therefore, a misbalance between TFC thickness and UV appears to exist for gymnasts. The misbalance could potentially alter axial wrist loading distribution of the wrist in gymnasts and provide the missing link to demonstrate a relationship between UV and distal radial physeal injury or wrist pain.

## Conflicts of Interest

No benefits in any form have been received or will be received related directly to this article.

## Supplementary Data

Figure S1 This supplementary material presents the scoring form that was developed in a prior study and used to perform MRI assessment.
