# Supplementary material for: The Influence of Early Gymnastic Exposure on the Triangular Fibrocartilage Complex in the Adolescent Wrist
Source: J Hand Surg Glob Online. 2026 Feb 24;8(3):100969. doi: 10.1016/j.jhsg.2026.100969 (PMC12973695; doi:10.1016/j.jhsg.2026.100969)
Supplement: Figure S1 — This supplementary material presents the scoring form that was developed in a prior study and used to perform MRI assessment. [file mmc1.pdf]

Supplementary Material. Score form used for triangular fibrocartilage complex assessment in healthy asymptomatic adolescents with illustrative images from young symptomatic gymnasts that were not included in the present study

**A WRIST POSITIONING**			
1.1 **Ulnar prestyloid recess position**<br>*Axial PD*	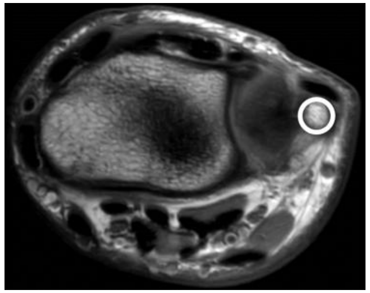<br>☐ Neutral	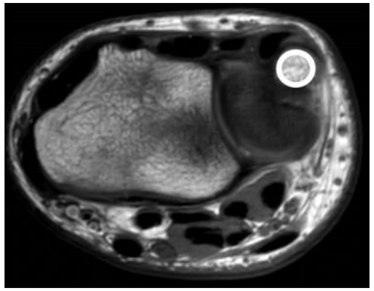<br>☐ Dorsal rotation (supinated)	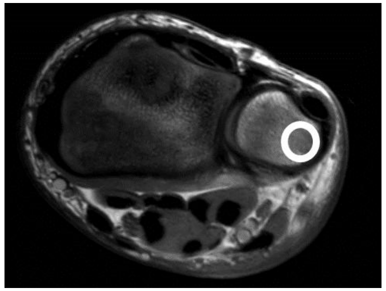<br>☐ Volar rotation (pronated)
**B TRIANGULAR FIBROCARTILAGE (TFC)**			
1.1 **TFC morphology – On the sagittal slice where the TFC is at its thinnest**<br>*Sagittal PD*	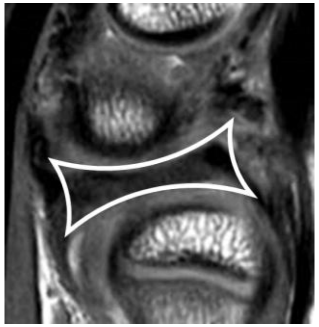<br>☐ Symmetrical biconcave disc	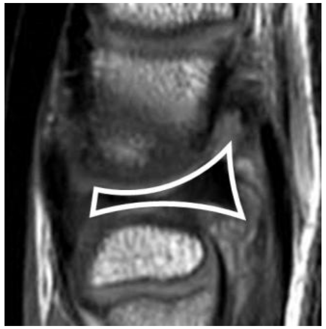<br>☐ Biconcave disc dorsal thicker than volar	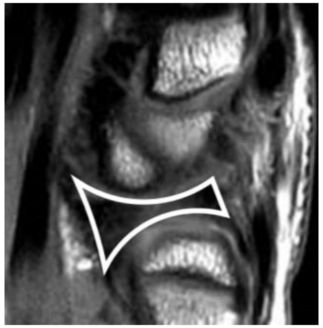<br>☐ Biconcave disc volar thicker than dorsal

1.2 **TFC morphology – On the <u>mid-coronal</u> slice where the TFC is at its thinnest**<br>*Coronal PD and PD SPAIR*	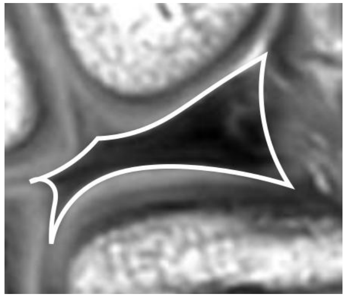<br>☐ Slightly radial tilted asymmetrical bowtie	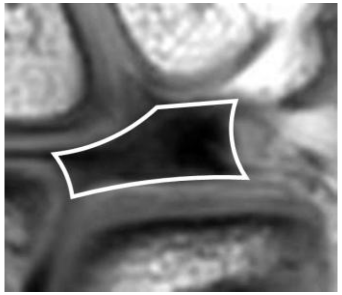<br>☐ Shorter, thicker and more horizontal structure	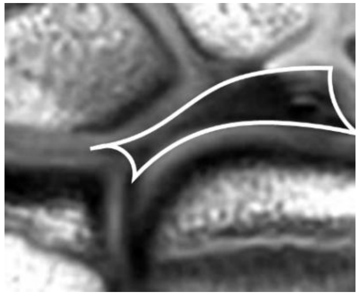<br>☐ Thinner and more stretched structure	
1.3 **TFC thickness – On the <u>mid-coronal</u> slice where the TFC is at its thinnest**<br>*Coronal PD and PD SPAIR*		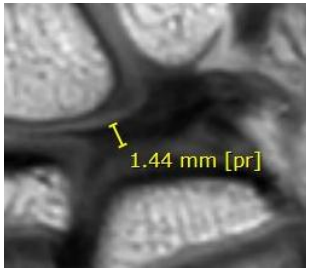<br><br>_ . _ _ mm at the thinnest point	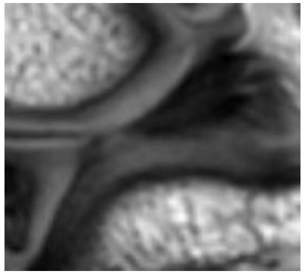<br>0.00 mm when cannot be measured	
1.4 **Homogeneity**<br>*Coronal PD and PD SPAIR*	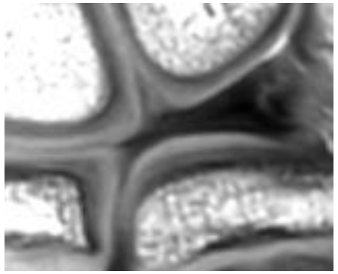<br>☐ Homogeneous hypointens <u>without</u> increased signal	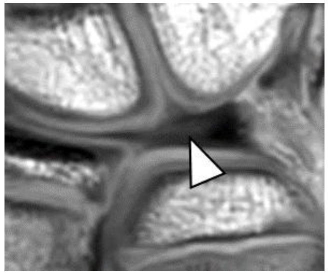<br>☐ Diffuse increased signal <u>not</u> extending to the joint surface	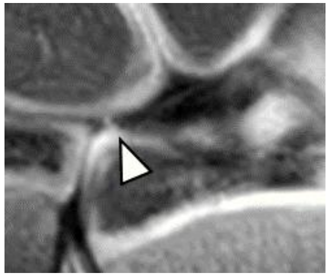<br>☐ Linear <u>vertical</u> increased signal with disruption of the disc	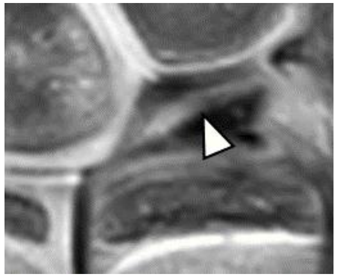<br>☐ Other

**C RADIOULNAR LIGAMENTS (RUL's)**			
1.1 **Homogeneity <u>dorsal</u> RUL – sagittal**<br>*Sagittal PD*	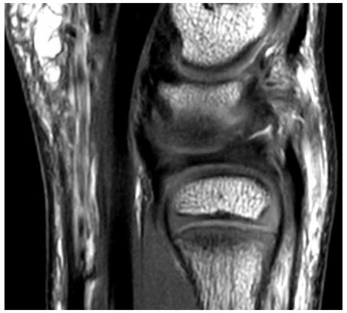<br>☐ Continuous with TFC	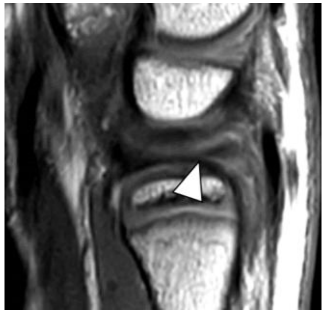<br>☐ Not continuous with TFC	☐ Not able to assess
1.2 **Homogeneity <u>volar</u> RUL – sagittal**<br>*Sagittal PD*	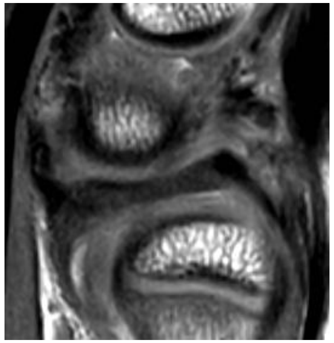<br>☐ Continuous with TFC	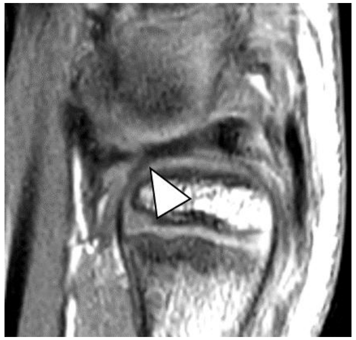<br>☐ Not continuous with TFC	☐ Not able to assess

1.3 **Fiber continuity <u>dorsal</u> RUL – axial**<br>*Axial PD and T2 SPAIR*	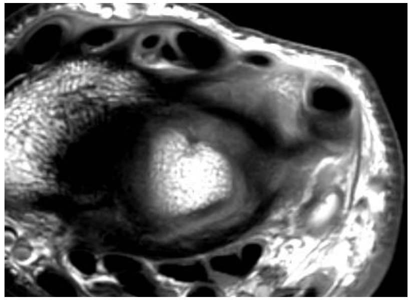<br>☐ Continuous fibers	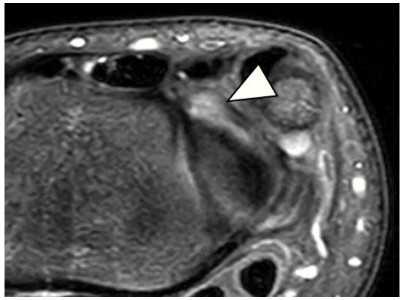<br>☐ Fiber disruption	☐ Not able to assess
1.4 **Fiber continuity <u>volar</u> RUL – axial**<br>*Axial PD and T2 SPAIR*	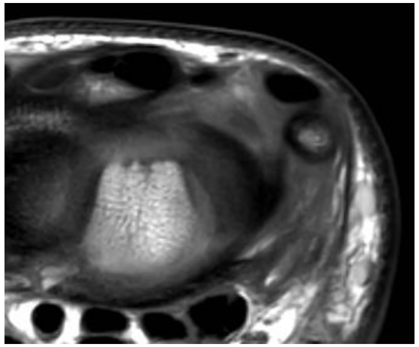<br>☐ Continuous fibers	☐ Fiber disruption	☐ Not able to assess
**D PROXIMAL (DEEP) AND DISTAL (SUPERFICIAL) LAMINA**			
1.1 **Homogeneity <u>proximal lamina</u>**<br>*Coronal PD and PD SPAIR / sagittal PD*	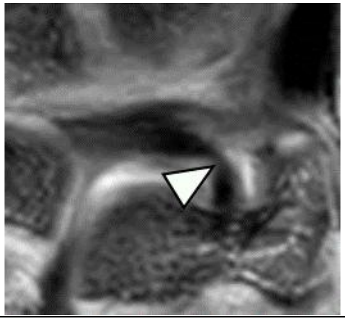	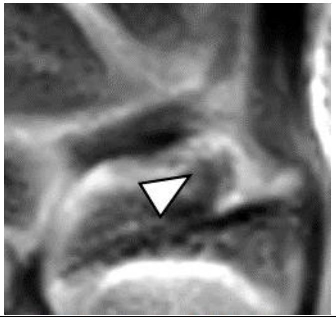	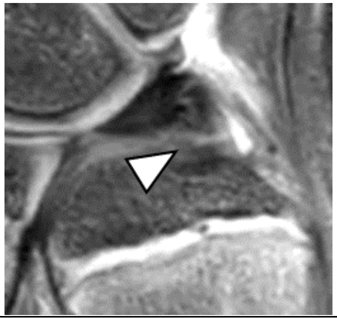

	☐ Homogeneous hypointens	☐ Diffuse lamination	☐ Not able to assess

<table>
<tr><td colspan="2">1.2 Homogeneity <u>distal lamina</u>
Coronal PD and PD SPAIR / sagittal PD</td><td colspan="2">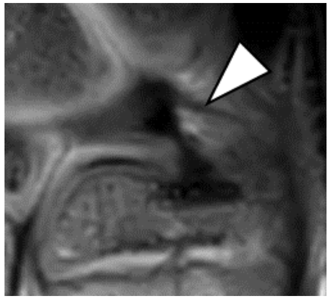
☐ Homogeneous hypointens</td><td colspan="2">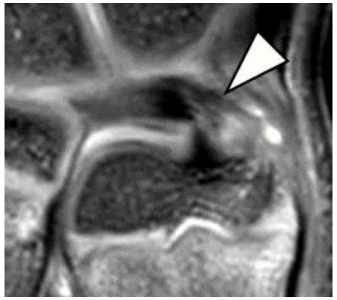
☐ Diffuse lamination</td><td colspan="2">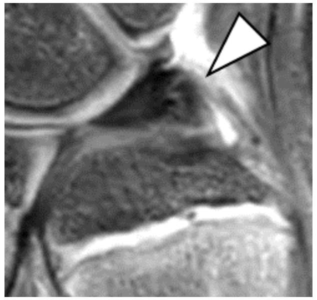
☐ Not able to assess</td></tr>
<tr><td colspan="8">E LIGAMENTUM SUBCRUENTUM</td></tr>
<tr><td colspan="2">1 Visibility
Coronal PD SPAIR</td><td></td><td colspan="2">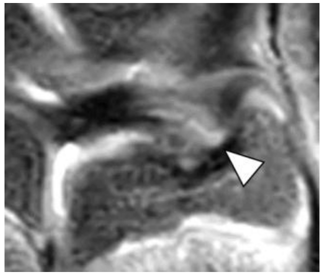
☐ Hyperintens signal between the proximal and distal lamina</td><td colspan="2">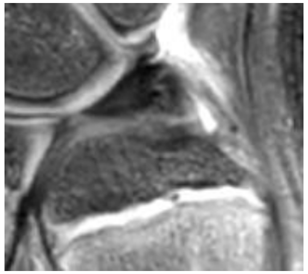
☐ Not visible</td><td></td></tr>
</table>

F	PRESTYLOID RECESS				
1	**Visibility**<br>*Coronal PD SPAIR*	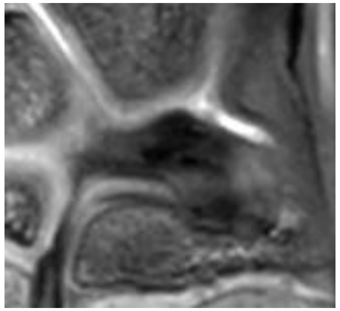<br>☐ Tubular shaped	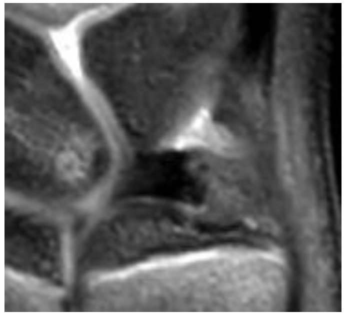<br>☐ Conical shaped	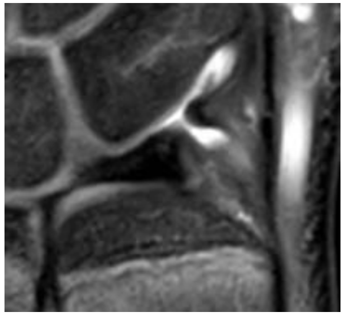<br>☐ Saccular shaped	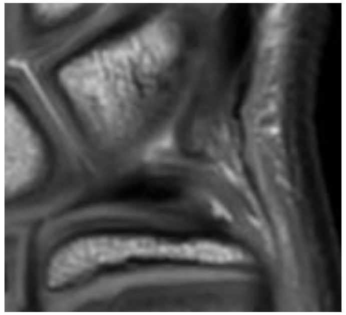<br>☐ Not visible

G	MENISCUS HOMOLOGUE			
1	**Visibility**<br>*Coronal PD en PD SPAIR*	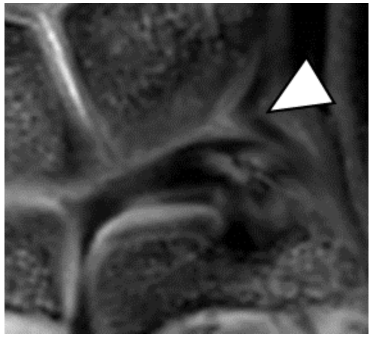<br>☐ Clearly delineated hypointensity	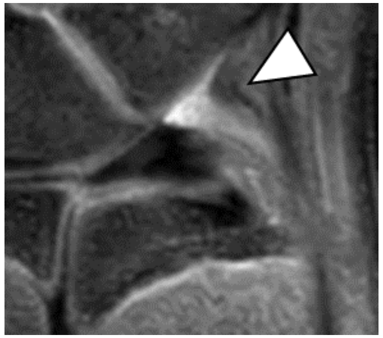<br>☐ Diffuse hypointensity	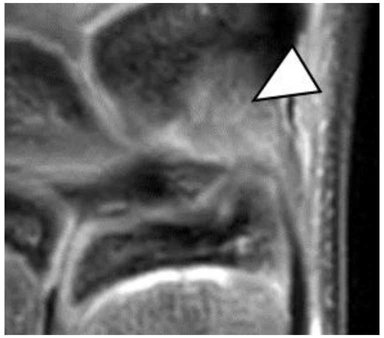<br>☐ Not visible due to diffuse hyperintensity

H	EXTENSOR CARPI ULNARIS (ECU)			
1.1 **ECU position in ulnar groove** *Axial PD and T2 SPAIR*	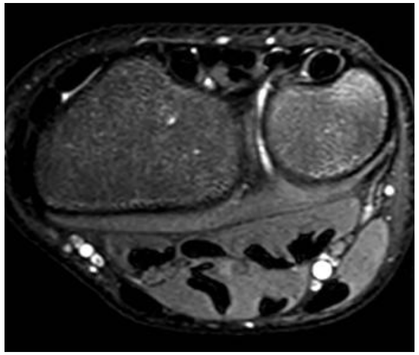 ☐ Completely within ECU groove	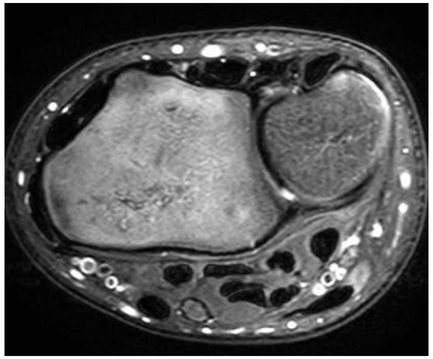 ☐ Partially within ECU groove	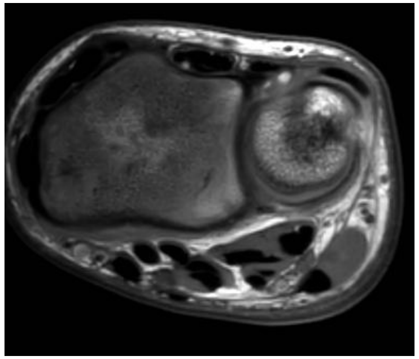 ☐ Completely outside ECU groove	
1.2 **<u>Peritendinous</u> signal intensity – from the ECU groove until distal extensor retinaculum** *Axial T2 SPAIR*		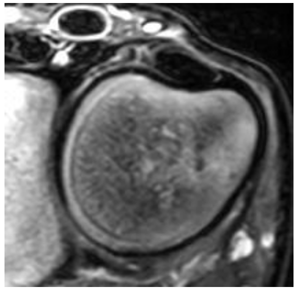 ☐ Hypointens or intermediate signal	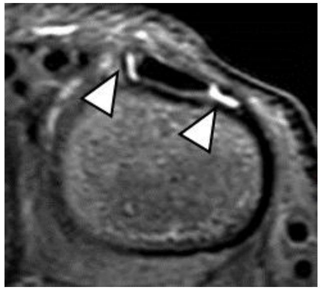 ☐ Focal increased signal	

1.3 **<u>Intratendinous</u> signal intensity – from the ECU groove until distal extensor retinaculum**<br>*Axial T2 SPAIR*		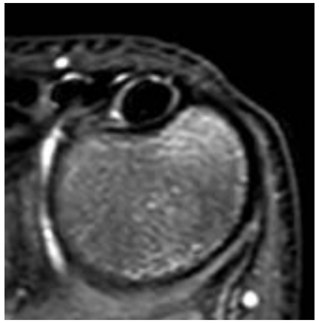<br>☐ Homogeneous hypointens	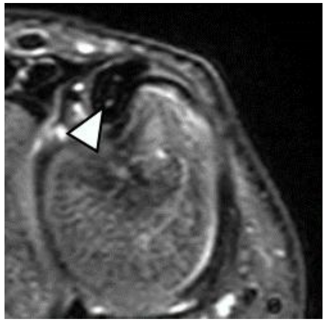<br>☐ Focal or linear increased signal	
1.4 **Location increased signal**<br>*Axial T2 SPAIR*	☐ Not applicable	☐ Proximal from the styloid process	☐ At the styloid process level	☐ Distal from the styloid process

I	DISTAL RADIOULNAR JOINT (DRUJ)		
1.1 **Effusion radioulnar**<br>*Coronal PD SPAIR and axial T2 SPAIR*	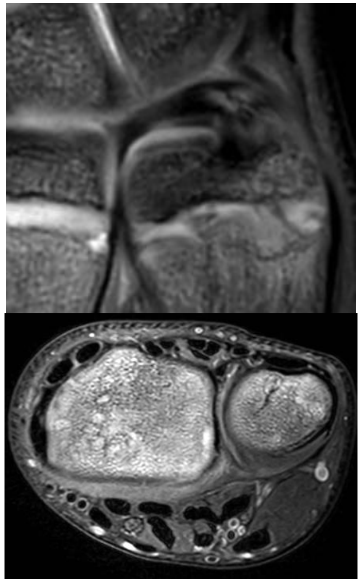<br>☐ Absent	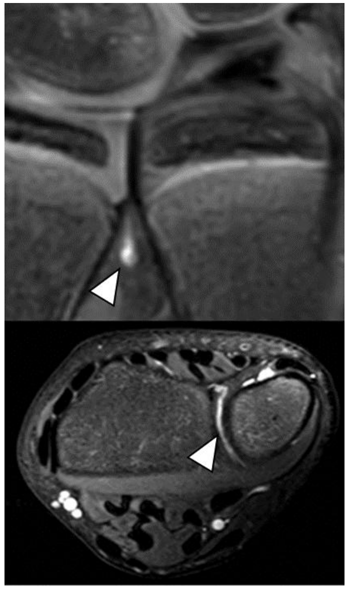<br>☐ Small amount	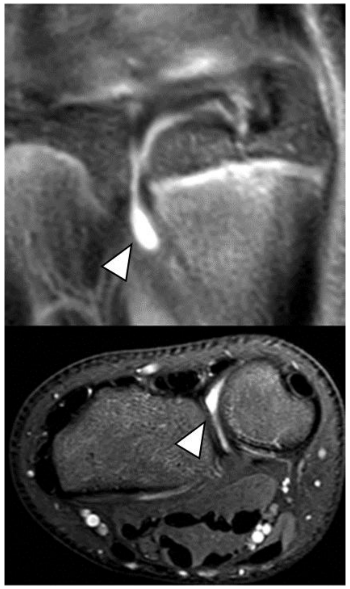<br>☐ Substantial amount

1.2 **Cysts – ulnar sided wrist**<br>*Coronal PD SPAIR and axial T2 SPAIR*	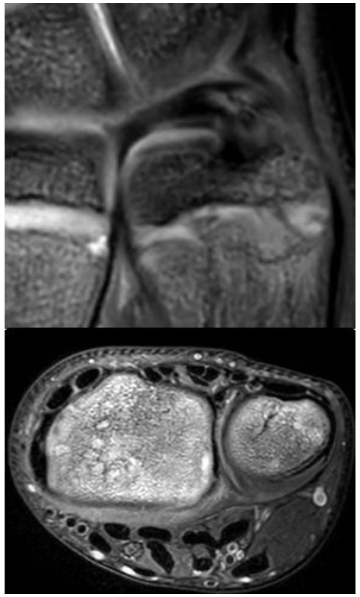<br>☐ Absent	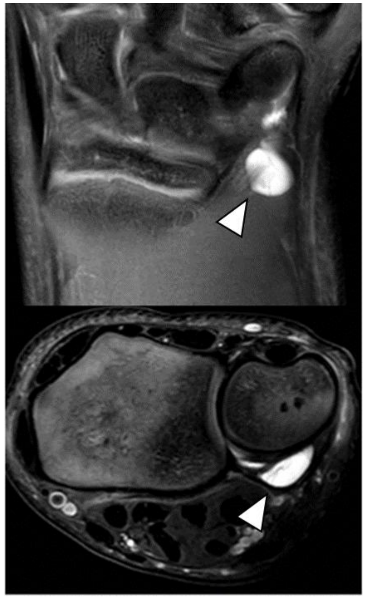<br>☐ Volar	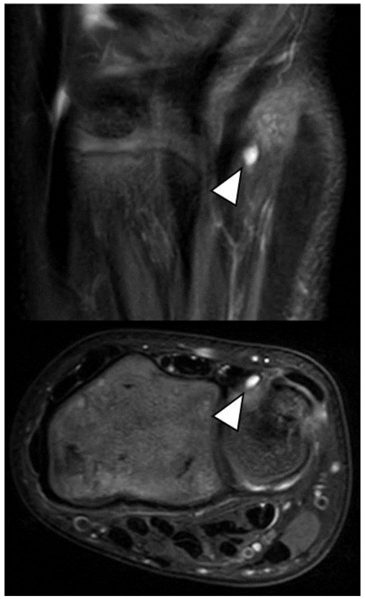<br>☐ Dorsal
**J PISOTRIQUETRAL JOINT (PTJ)**			
1.1 **PTJ effusion**<br>*Coronal PD SPAIR*	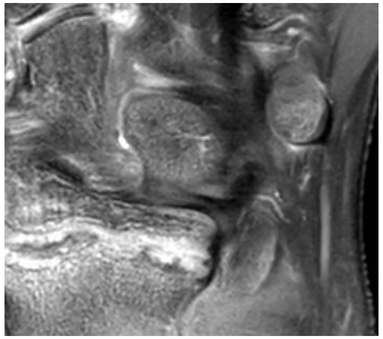<br>☐ Absent	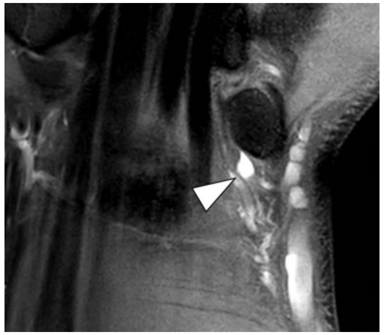<br>☐ Small amount	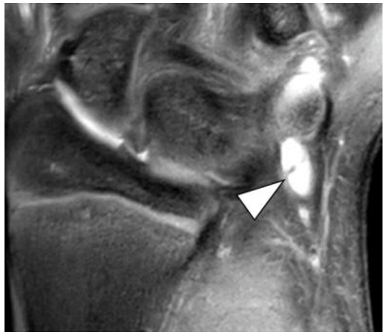<br>☐ Substantial amount

K	OTHER RELEVANT FINDINGS
